# Evaluation of Domestication Loci Associated with Awnlessness in Cultivated Rice, *Oryza sativa*

**DOI:** 10.1186/s12284-020-00386-4

**Published:** 2020-04-28

**Authors:** Yaddehige Priya Jayantha Amarasinghe, Rie Kuwata, Akinori Nishimura, Phuong Dang Thai Phan, Ryo Ishikawa, Takashige Ishii

**Affiliations:** 1 Graduate School of Agricultural Science, Kobe University, 1-1 Rokkodai, Nada-ku, Kobe, 657-8501 Japan; 2 Present address: Research Institute for Biotechnology and Environment, Nong Lam University, Ho Chi Minh, Vietnam

**Keywords:** Awn, Awnlessness, Domestication, Gene interaction, *Oryza rufipogon*, QTL mapping, Rice

## Abstract

**Background:**

Awns are bristle-like organs at the tips of the glumes. Wild rice has maintained long awns for successful seed propagation through seed dispersal. Seed awning is an interesting trait in rice domestication. Long awns might have been beneficial for seed gatherers in the initial phase of domestication; however, awnless phenotypes were preferably selected in a later phase with non-seed-shattering plants. Investigation of domestication loci associated with awnlessness in cultivated rice will be useful in clarifying the process and history of rice domestication.

**Results:**

Quantitative trait locus (QTL) analysis for seed awning was carried out using a BC_3_F_2_ population between *Oryza sativa* IR36 (a cultivated donor parent with awnless phenotype) and *O. rufipogon* W630 (a wild recurrent parent with awns). As a result, two QTLs on chromosome 4 (corresponding to *An-1* and *LABA1*) and one on chromosome 2 (designated as *qAWNL2*) were detected. Gene interaction among three seed-awning QTLs were further examined with the plants having eight different combinations of homozygous genotypes. Their awn length variation indicated that the IR36 alleles at these loci had the additive awnlessness effects in the genetic background of wild rice. The shortest awn length was observed for the plants having IR36 homozygous alleles at all loci, giving about 75% reduction in awn length. By the fine mapping, the candidate region of the novel *qAWNL2* locus was delimited in a 157.4-kb region containing 22 predicted genes in Nipponbare genome.

**Conclusions:**

QTL analysis revealed that three loci, *An-1, LABA1* and *qAWNL2*, were mainly responsible for the awnlessness of *O. sativa* IR36. In the wild genetic background, loss-of-function alleles at three awning loci showed additive effects on length reduction. In rice domestication, awnless forms may be gradually generated through the accumulation of mutations at awning loci.

## Background

Crop domestication which formed the basis for agriculture, has been considered a major turning point in human history (Fuller and Allaby 2009). In the transition from gathering to cultivation, early farmers selected wild plants with useful genetic modifications and developed improved populations with desirable traits (Vaughan et al. 2007). In cereals, non-seed-shattering behavior is a conspicuous trait distinguishing wild and cultivated forms, and an important adaptation syndrome resulting from automatic selection through harvesting (Harlan et al. 1973).

Rice (*Oryza sativa* L.) is a major food crop and a primary food source for approximately half of the world’s population (Zeigler and Barclay 2008). This crop was domesticated from the Asian wild species, *O. rufipogon* Griff (Oka 1988). Wild rice possesses several propagation-related traits, such as prostrate growth, seed shattering, open panicle structure, seed awning, and the ability to outcross. A reduction in seed shattering was a key trait for the emergence of cultivated rice because it directly enhanced the collection efficiency for ancient seed gatherers (Harlan et al. 1973).

In rice, three natural variants in the seed-shattering loci (*sh4, qSH1* and *qSH3*) have been reported (Li et al. 2006; Konishi et al. 2006; Htun et al. 2014). In the genetic background of cultivated rice, the wild functional alleles at two major loci, *sh4* and *qSH1*, show seed-shattering effects. However, in the genetic background of wild rice, the cultivated alleles at either locus do not lead to non-shattering phenotypes (Ishikawa et al. 2010; Htun et al. 2014). This suggests that wild rice has other minor genes associated with seed shattering. Non-shattering plants are not easily identified in wild rice populations. Previously, we found that a simple morphological change in panicle shape (from open to closed), controlled by a single locus of *SPR3*, led to a reduction in seed shedding in wild rice (Ishii et al. 2013). In wild plants with closed panicles, the upper mature seeds are temporarily retained by the support of long awns from lower immature seeds. Since this panicle shape change results in higher seed collection efficiency (Ishii et al. 2013), seed awning may have been a beneficial trait for seed gatherers in the initial phase of domestication. Interestingly, long awns on closed panicles also hinder free exposure to anthers and stigmas during the flowering stage. This may enhance self-pollination and the accumulation of recessive alleles at the seed-shattering loci. After the emergence of non-seed-shattering plants, seed awning may have become an undesirable trait because long awns disturb seed harvesting and handling (Ishii and Ishikawa 2018).

Awns are bristle-like organs at the tips of the glumes. Wild rice has retained long awns for successful seed propagation through seed dispersal. There are three major loci, *An-1*, *LABA1* and *RAE2*, for seed awning in wild rice (Luo et al. 2013; Hua et al. 2015; Bessho-Uehara et al. 2016). At these loci, wild functional alleles worked for seed awning in cultivated rice, but cultivated loss-of-function alleles did not contribute much to awn length reduction in wild rice (Ikemoto et al. 2017). These allele effects are similar to those which control seed shattering in the genetic background of wild rice, indicating that other minor loci are associated with these wild traits.

Seed awning is an interesting trait in rice domestication. Long awns might have been beneficial for seed gatherers in the initial phase of domestication; however, awnless phenotypes were preferably selected in a later phase with non-seed-shattering plants. In this study, first, a quantitative trait locus (QTL) analysis for awnlessness was carried out using a wild backcrossed population between *O. sativa* IR36 and *O. rufipogon* W630. Then, gene interactions at these loci were examined along with causal gene estimation, in order to clarify the genetic mechanisms of awnlessness in cultivated rice.

## Materials and Methods

### Plant Materials

**Fig. 1:**
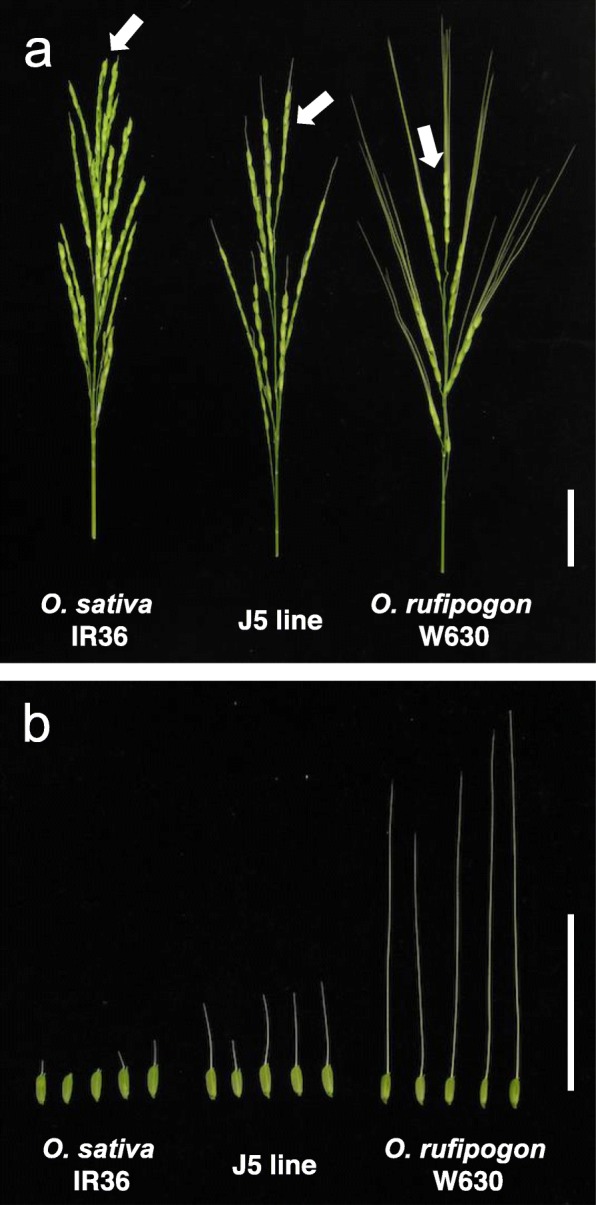
Panicle and spikelet morphology of the *O. sativa* IR36, J5 line and *O. rufipogon* W630. **a** Whole panicles. Arrows indicate top primary branches in the panicles. **b** Spikelets on the top primary branches. The 1st to 5th spikelets are arranged from left to right. Scale bar: 5 cm

**Fig. 2:**
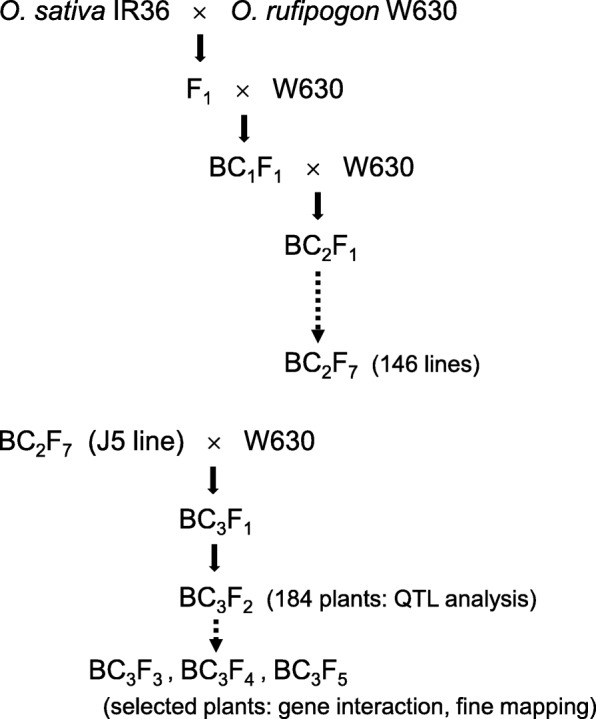
Pedigree of backcross recombinant inbred lines between *O. sativa* IR36 and *O. rufipogon* W630. A single line of J5 was further backcrossed with W630, and their BC_3_ progenies were used for the genetic analysis

A long-awned accession of *O. rufipogon* W630 and an awnless cultivar of *O. sativa* IR36 were used in this study (Fig. 1). This wild accession was provided by the National Institute of Genetics, Japan. IR36 is one of the most popular Indica cultivars released from International Rice Research Institute (Khush and Virk 2005). A single plant of *O. sativa* IR36 was crossed three times with *O. rufipogon* W630, and 146 backcross recombinant inbred lines (BILs) were developed at the BC_2_F_7_ generation by the single-seed-descendant method (Fig. 2). Since they had the genetic background of wild rice, all the BILs had awned seeds. Among them, a single line J5 had the shortest awns and was selected to examine the awning loci (Fig. 1). The J5 line was crossed with W630, and 184 BC_3_F_2_ plants were produced (Fig. 2). They were phenotypically evaluated in the paddy field for the awn length variation. The progenies from the selected BC_3_F_2_ plants were further used for the evaluation of gene interaction and fine mapping.

### Evaluation of Awn Length

In wild rice, awn length varies according to the spikelet position in the panicle (Fig. 1a) (Ikemoto et al. 2017), and the seeds shatter in the mature stage. Therefore, the upper 1st to 5th spikelets on the top primary branch in a panicle were collected in the flowering stage, and their awn lengths were measured (Fig. 1b). The averages of five panicles were calculated for each plant.

### QTL Analysis for Seed Awning

Total DNA was extracted from the J5 line at the BC_2_F_7_ generation by the potassium acetate method (Dellaporta et al. 1983), and the marker genotypes were surveyed at 159 simple sequence repeat (SSR) loci across 12 rice chromosomes. PCR was carried out in a 25-μl reaction, and the amplified products were electrophoresed in 4% polyacrylamide denaturing gel. The microsatellite banding patterns were visualized using the silver staining method (Panaud et al. 1996). QTL analysis for seed awning was further carried out with 38 SSR markers segregating in the BC_3_F_2_ population (Fig. 2). In addition, two markers of An1-D and LABA1-D, which detect causal mutations at *An-1* and *LABA1* loci, respectively, were included in the analysis (Additional file 1: Table S1). A putative QTL was estimated by single marker analysis based on the logarithm of odds (LOD) score using QGene ver.3.0 (Nelson 1997).

### Evaluation of Gene Interaction at Seed-Awning Loci

At a single locus, two homozygous genotypes (IR36 or W630 homozygotes) are expected in the BILs. Since three QTLs were detected for seed awning, a total of eight combinations of homozygous genotypes were examined for gene interaction on seed awning. In each combination, five BC_3_F_3_ plants were produced from the different BC_3_F_2_ plants having IR36 chromosomal segments at specific awning loci (Fig. 2). They were grown in the paddy field, and five panicles were collected from each plant at the heading stage. Their awn lengths were measured based on the spikelet positions, and the averages were compared among eight combinations of genotypes together with the parental lines by Tukey’s significance test.

### Fine Mapping of Seed-Awning Locus on Chromosome 2

The BC_3_F_3_ progenies were first surveyed based on the marker genotypes at two known seed-awning loci, and a single plant was selected. This plant had loss-of-function alleles at *An-1* and *LABA1* loci and the heterozygous chromosomal segment covering the putative QTL on chromosome 2. It was selfed and the BC_3_F_4_ plants possessing recombination within the putative QTL region were selected. Progeny test for seed awning was carried out using BC_3_F_5_ plants derived from the critical recombinants (Fig. 2). The average awn lengths of five plants were compared between two homozygous genotypes by t-test.

## Results

### Awn Phenotypes and Marker Genotypes of the J5 Backcross Recombinant Inbred Line

The J5 line showed the shortest awns among 146 BILs at BC_2_F_7_ generation. Compared with the wild recurrent parent of *O. rufipogon* W630, the awn length was much reduced for all the spikelets in 1st to 5th positions; 73.5 ~ 81.1% length reduction (Fig. 1, Table 1). The chromosomal constitution was surveyed with 159 molecular markers, and 38 marker loci were found to have homozygous IR36 alleles. They were located on 11 chromosomal segments introgressed into the wild rice from IR36 (Additional file 2: Figure S1).

**Table 1:** Mean awn lengths (mm) and standard deviation observed for the parental lines, *O. sativa* IR36, *O. rufipogon* W630 and the J5 line (*n* = 5)

Line	Spikelet position				
	1st	2nd	3rd	4th	5th
*O. sativa* IR36	1.4 ± 0.3	0.0 ± 0.0	0.0 ± 0.0	0.1 ± 0.1	0.5 ± 0.4
*O. rufipogon* W630	78.2 ± 5.1	66.7 ± 2.5	85.1 ± 3.7	97.1 ± 3.5	105.4 ± 3.1
J5 line (% length reduction) ^a^	20.7 ± 0.9	12.6 ± 0.3	17.0 ± 0.4	20.6 ± 1.6	22.2 ± 1.8
	(73.5%)	(81.1%)	(80.0%)	(78.8%)	(78.9%)

^a^Percentage of length reduction compared to *O. rufipogon* W630

### QTL Analysis for Seed Awning

In the BC_3_F_2_ population between the J5 line and *O. rufipogon* W630, discontinuous distributions of awn lengths were observed for all the spikelets in the 1st to 5th positions (Additional file 2: Figure S2). QTL analysis was carried out with these awn data and the marker genotypes at the 38 loci located on the introgressed segments. As a result, one weak peak was detected on chromosome 2, while a strong peak was detected on chromosome 4. Although the allele effects at the putative QTL on chromosome 2 was small, the peaks were constantly observed for the awn lengths in the 1st to 5th spikelet positions (LOD = 1.9 ~ 3.2, PV = 4.6 ~ 7.7%) (Table 2). We named the locus *qAWNL2* (*QTL for awn length on chromosome 2*). The QTL region on chromosome 4 contained two major awning loci of *An-1* and *LABA1*, and IR36 was previously reported to have loss-of-function alleles at both loci (Luo et al. 2013; Hua et al. 2015). Therefore, two additional markers of An1-D and LABA1-D detecting these causal mutations were included in the analysis (Additional file 1: Table S1). These markers showed two QTL peaks with high LOD values (An1-D: 18.6 ~ 22.8, LABA1-D: 24.4 ~ 35.0) in the region (Table 2), indicating that the QTLs on chromosome 4 were identical to *An-1* and *LABA1*.

**Table 2:** Putative QTL locations for awnlessness detected in BC_3_F_2_ population between *O. rufipogon* W630 and *O. sativa* IR36

Chr.	Locus	Marker ^a^	Source ^b^	Spikelet position ^c^	LOD	PV ^d^ (%)
2	*qAWNL2*	RM1211	IR36	1st	3.2	7.7
				2nd	2.2	5.3
				3rd	2.1	5.0
				4th	2.0	4.9
				5th	1.9	4.6
4	*An-1*	An1-D	IR36	1st	20.3	39.9
				2nd	18.9	37.7
				3rd	18.6	37.2
				4th	21.0	40.9
				5th	22.8	43.5
4	*LABA1*	LABA-D	IR36	1st	24.4	45.7
				2nd	35.0	58.4
				3rd	33.4	56.6
				4th	28.9	51.5
				5th	26.7	48.8

^a^Marker giving high LOD by single point analysis

^b^Source of the allele having awnlessness effect

^c^Spikelet position of awn length data

^d^Percentage of the phenotypic variance explained by the QTL

### Gene Interaction among Three Seed-Awning QTLs

**Fig. 3:**
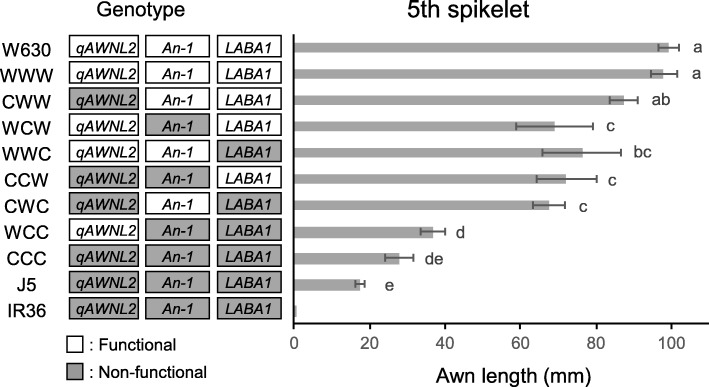
Awn lengths (means ± SD, *n* = 5) of the 5th spikelets of eight homozygous genotypes in the wild genetic background. These are compared together with the parental accessions of *O. rufipogon* W630 and the J5 line. Average awn lengths of *O. sativa* IR36 are shown for reference. Eight genotypes were designated by three-letter combinations of W or C in genotypic order at *qAWNL2*, *An-1* and *LABA1*, where W and C indicate wild and cultivated homozygous alleles, respectively. White and gray boxes with the locus names beside the genotypes indicate functional (wild) and non-functional (cultivated) homozygous alleles, respectively. Means labeled with different letters are significantly different among eight genotypes and two parental lines (Tukey’s test, *P* < 0.05)

In this study, high LOD peaks at *qAWNL2*, *An-1* and *LABA1* were detected for the awn lengths in all the spikelet positions. At these loci, the IR36 alleles had the awnlessness effect in the wild genetic background. Gene interaction among three seed-awning QTLs were further examined with the BC_3_F_3_ plants having eight different combinations of homozygous genotypes. Here, the eight genotypes were designated by adding three letters of W or C in genotypic order at *qAWNL2*, *An-1* and *LABA1*, where W and C indicate wild and cultivated homozygous alleles, respectively. In each combination, five plants were selected from the different BC_3_F_2_ plants based on the linked marker genotypes. Their awn lengths were measured based on the spikelet positions and compared together with the parental accessions (Additional file 2: Figure S3). The average awn length of the 5th spikelet of WWW plants (having wild homozygous alleles at all three loci) was almost the same as that of wild parent W630, whereas a slight length reduction (ca. 12%) was observed for the CWW plants with IR36 homozygous alleles only at *qAWNL2* (Fig. 3). The four genotypes WCW, WWC, CCW, and CWC with IR36 homozygous alleles at either *An-1* or *LABA1*, showed significant length reduction; around 1/3 shorter than that of W630. The WCC plants with IR36 homozygous alleles at both *An-1* and *LABA1* had much shorter awns with about a 2/3 reduction in length. The shortest awn length was observed for the CCC plants having IR36 homozygous alleles at all loci, but it was slightly longer than that of the J5 line. Similar length variation and gene interaction were also detected for the awns of other spikelets in the 1st to 4th positions (Additional file 2: Figure S4). The average awn lengths and percentages of reduction for all the genotypes are summarized in Table 3.

**Table 3:** Average lengths and reduction percentages of the awns in the 1st to 5th spikelet positions of eight homozygous genotypes and the parental accessions of *O. rufipogon* W630 and the J5 line

Position	W630	Homozygous genotype ^a^								J5
		WWW	CWW	WCW	WWC	CCW	CWC	WCC	CCC	
Awn length (mm)										
1st	71.5	70.9	59.8	54.0	57.9	53.0	51.0	29.8	24.4	16.5
2nd	59.3	62.8	52.8	40.4	39.6	36.8	36.6	14.9	9.9	8.1
3rd	75.1	78.7	67.9	53.2	51.9	52.1	47.2	22.2	15.5	11.3
4th	89.3	89.1	78.5	62.8	70.0	65.0	60.3	30.7	23.1	16.6
5th	99.3	98.1	87.4	69.2	76.4	72.2	67.5	36.7	27.8	17.5
Perecentage of reduction ^b^										
1st	0	0.8	16.4	24.5	19.0	25.8	28.7	58.4	65.9	76.9
2nd	0	−5.8	10.9	31.9	33.2	38.0	38.3	74.9	83.3	86.4
3rd	0	−4.8	9.6	29.2	30.9	30.6	37.2	70.5	79.3	84.9
4th	0	0.2	12.1	29.7	21.6	27.2	32.5	65.6	74.2	81.4
5th	0	1.2	12.0	30.3	23.0	27.2	32.0	63.0	72.0	82.4
Average ^c^	0	−1.7	12.2	29.1	25.5	29.8	33.8	66.5	74.9	82.4
	a	a	b	c	c	c	c	d	de	e

^a^Eight genotypes were designated by three-letter combinations of W or C in genotypic order at *qAWNL2*, *An-1* and *LABA1*, where W and C indicate wild and cultivated homozygous alleles, respectively

^b^Length reduction compared to W630

^c^Overall averages given with different letters are significantly different among the eight genotypes and two parental lines (Tukey’s test, *P* < 0.05)

### Fine Mapping of Seed-Awning Locus, *qAWNL2*

**Fig. 4:**
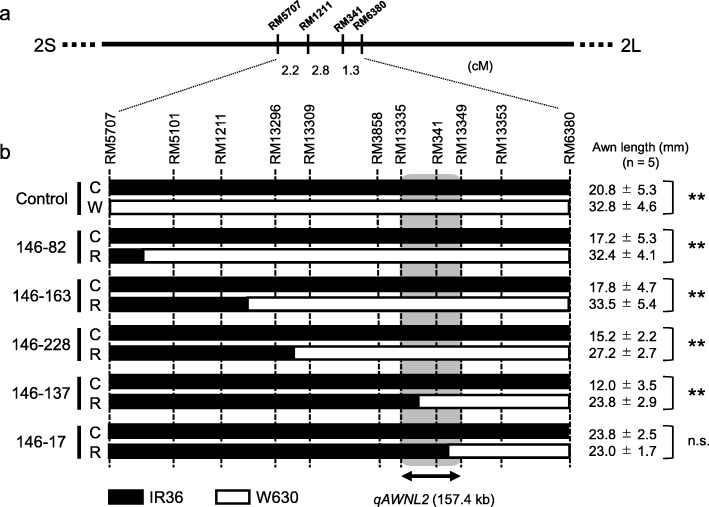
Fine mapping of *qAWNL2*. **a** Molecular markers between RM5707 and RM6380 on chromosome 2 were used to detect recombinants near the *qAWNL2* locus. **b** Graphical genotypes of BC_3_F_5_ lines derived from five critical recombinant BC_3_F_4_ plants (nos. 146–82, − 211, − 228, − 137 and − 17) in the candidate region of *qAWNL2*. C, W and R: Homozygous IR36, W630 and recombinant chromosomes, respectively. Awn lengths (means ± SD, n = 5) of the 5th spikelets of the BC_3_F_5_ lines are shown on right. ** and n.s.: significant at *P* < 0.01 and not significant, respectively. The *qAWNL2* locus was estimated in the 157.4-kb region between RM13335 and RM13349

Although the allele effect at *qAWNL2* was weaker than those at *An-1* and *LABA1*, awn length difference was clearly detected between the WCC and CCC plants. Therefore, a single BC_3_F_3_ plant (plant no. 146) with cultivated loss-of-function alleles at *An-1* and *LABA1* and the heterozygous chromosomal segment containing the *qAWNL2* locus was further selfed for fine mapping. The plants displaying recombination within the putative QTL region were surveyed among 580 BC_3_F_4_ plants using a pair of markers, RM5707 and RM6380, and the recombination positions were examined with nine SSR markers located in the putative region (Fig. 4, Additional file 1: Table S1). At the BC_3_F_5_ generation, the progeny tests were carried out between the lines with the homozygous IR36 and recombinant chromosomal segments derived from the representative recombinants. As a result, two critical BC_3_F_4_ plants, nos. 146–137 and 146–17, were found to have recombination between RM13335 and RM341 and between RM341 and RM13349, respectively (Fig. 4). In the BC_3_F_5_ plants from no. 146–137, the average awn length of the 5th spikelet of the plants with homozygous recombinant chromosomes (R: 23.8 mm) was significantly longer than that with homozygous IR36 chromosomes (C: 12.0 mm) (t-test, *P* < 0.01). The significant length differences (*P* < 0.01) were also observed between the two homozygous groups for the 1st to 4th positions of the spikelets (Additional file 1: Table S2). In contrast, no significant differences in awn lengths were observed for any positions of spikelets between two homozygous BC_3_F_5_ genotypes (C and R) derived from no. 146–17 (Fig. 4, Additional file 1: Table S2). These progeny tests indicate that the candidate region of *qAWNL2* was within a 157.4-kb region between RM13335 and RM13349. In this region, a total of 22 genes were predicted based on the sequence annotation for *O. sativa* Japonica Nipponbare by RAP-DB (Additional file 1: Table S3) (Sakai et al. 2013). Further investigations are necessary to specify the gene of *qAWNL2*.

## Discussion

### Evaluation of Seed Awning

Seed awning is one of the wild-specific traits in rice. In order to detect the responsible loci for this trait, several QTL analyses have been carried out using interspecific populations between wild accessions and cultivars (Xiong et al. 1999; Cai and Morishima 2002; Thomson et al. 2003; Gu et al. 2005). In these studies, seed awning was evaluated based on the presence/absence of awns or the average awn length of seeds. The former evaluation is unstable because the awn phenotype usually shows a continuous distribution in the interspecific populations. The latter evaluation does not consider the awn length variation caused by the spikelet position in the panicle. Therefore, only the major loci explaining high phenotypic variance could be detected in these analyses. Their wild alleles with strong awning effects were further investigated in the genetic background of cultivated rice, and three genes at *An-1*, *LABA1* and *RAE2*, were identified (Luo et al. 2013; Hua et al. 2015; Bessho-Uehara et al. 2016). However, other minor loci were not detected because of the rough evaluation methods. In general, awn length varies according to the position of the spikelet in the panicle (Table 1), and the numbers of seeds and primary/secondary branches are different among the plants. Therefore, in this study, awn lengths were compared between spikelets in the same position on the top primary branch for the precise evaluation. As a result, a minor locus of *qAWNL2* was detected in the QTL analysis, and the chromosomal location was successfully estimated by the progeny test.

### Allele Effects of Seed Awning in the Genetic Background of Wild Rice

The awning effects of wild functional alleles at major loci are easy to detect in the genetic background of cultivated rice. Conversely, the cultivated loss-of-function alleles are difficult to evaluate in the wild genetic background where many other wild awning genes are expressed. Therefore, cultivated allele effects of awnlessness were examined with different combinations of genotypes at three awning loci, *An-1, LABA1* and *qAWNL2*. For all the genotypes, awn length variations in the panicles were observed, but their length reduction ratios were similar for the 1st to 5th spikelets (Table 3). The significant differences among the overall averages (1st to 5th) of length reduction revealed by Tukey’s test suggest that genotype combinations at three loci have a wide influence on the awn length in the panicles.

The overall averages of length reduction of the CWW, WCW and WWC genotypes were 12.2%, 29.1% and 25.5%, respectively. These ratios directly explain the awnless effect of loss-of-function allele at either locus. Namely, the IR36 alleles at two major loci, *An-1* and *LABA1*, reduced the awn length of W630 by 25–30%, while those at *qAWNL2* had a weak effect (12.2% reduction). Among the IR36 alleles at these loci, additive effects were clearly observed for length reduction. The CCW and CWC genotypes showed slightly higher reduction ratios than the WCW and WWC genotypes, respectively. A 2/3 reduction ratio (66.5%) was observed for the awn length of the WCC plants. The CCC plants accumulating loss-of-function mutations at all loci had the highest length reduction of 74.9%, corresponding to about 90% of awnlessness of the J5 line (82.4% reduction). Since the J5 line still had short awns, the complete awnlessness in IR36 may be generated by additional accumulation of loss-of-function mutations at minor loci.

### Awnlessness in the Cultivated Rice

Until now, three major loci, *An-1*, *LABA1* and *RAE2*, have been reported for seed awning, and their wild alleles have a strong awn-elongation effect (Luo et al. 2013; Hua et al. 2015; Bessho-Uehara et al. 2016). In this study, a reduction of about 66% in awn length was explained by the loss-of-function alleles at two major loci of *An-1* and *LABA1*, and a cumulative 75% reduction was attained with the additional allele effect at *qAWNL2*. Interestingly, no QTL was detected around *RAE2* region on chromosome 8. This may be due to the fact that the IR36 allele is functional at *RAE2*. Previously, we examined awnlessness using the backcross population between *O. rufipogon* W630 and *O. sativa* Nipponbare (Ikemoto et al. 2017). Two major loci, *An-1* and *RAE2*, were detected by QTL analysis in the genetic background of wild rice. No QTL was found around *LABA1* locus, because Nipponbare has the functional allele at *LABA1* (Hua et al. 2015). These results indicate that the awnless characters of IR36 and Nipponbare are achieved by loss-of-function alleles at two of three major loci and some minor loci. Namely, the complete suppression of awn elongation may not require the mutations at all three major loci, *An-1*, *LABA1* and *RAE2*. Probably, certain combinations of mutations are associated with the awnless phenotypes of cultivars.

## Conclusions

This study investigates the domestication loci associated with awnlessness in cultivated rice, *O. sativa*. QTL analysis revealed that three loci, *An-1, LABA1* and *qAWNL2*, were mainly responsible for the awnlessness of *O. sativa* IR36. In the initial phase of rice domestication, long awns may be beneficial for seed gatherers, because they enhance the collection efficiency. The selection for shorter awns may have started after the emergence of non-seed-shattering plants. In the wild genetic background, loss-of-function alleles at three awning loci showed additive effects on length reduction. Therefore, awnless forms may be gradually generated through the accumulation of mutations at awning loci. Once a series of key awning loci are determined, the combination of mutations among awnless cultivar groups will be useful in clarifying the process and history of rice domestication in detail.

## Supplementary information

**Additional file 1: Table S1.** Additional molecular markers used for QTL analysis and fine mapping. **Table S2.** Average awn lengths (mm) and standard deviation observed for the homozygous genotypes derived from the recombinants (*n* = 5). **Table S3.** List of genes annotated in a 157.4-kb candidate region of *qAWNL2*, by the RAP-DB.

**Additional file 2: Figure S1.** Graphical genotype of the J5 backcross recombinant inbred line. **Figure S2.** Frequency distributions of awn lengths in the 1st to 5th spikelet positions in BC_3_F_2_ population between *O. rufipogon* W630 and the J5 line. **Figure S3.** Spikelet morphology of the eight genotypes of BC_3_F_3_ plants together with *O. rufipogon* W630, J5 line and *O. sativa* IR36. **Figure S4.** Awn lengths (means ± SD) of the 1st to 4th spikelets of eight homozygous genotypes in the wild genetic background.

## Data Availability

All data supporting the conclusions described here are provided in tables and figures.

## Abbreviations

BIL: Backcross recombinant inbred line
LOD: Logarithm of odds
QTL: Quantitative trait locus
SSR: Simple sequence repeat
