# Supplementary material for: Evaluation of Domestication Loci Associated with Awnlessness in Cultivated Rice, Oryza sativa
Source: Rice (N Y). 2020 Apr 28;13:26. doi: 10.1186/s12284-020-00386-4 (PMC7188743; doi:10.1186/s12284-020-00386-4)
Supplement: Supplementary file 2 — Additional file 2: Figure S1. Graphical genotype of the J5 backcross recombinant inbred line. Figure S2. Frequency distributions of awn lengths in the 1st to 5th spikelet positions in BC3F2 population between O. rufipogon W630 and the J5 line. Figure S3. Spikelet morphology of the eight genotypes of BC3F3 plants together with O. rufipogon W630, J5 line and O. sativa IR36. Figure S4. Awn lengths (means ± SD) of the 1st to 4th spikelets of eight homozygous genotypes in the wild genetic background. [file 12284_2020_386_MOESM2_ESM.pdf]

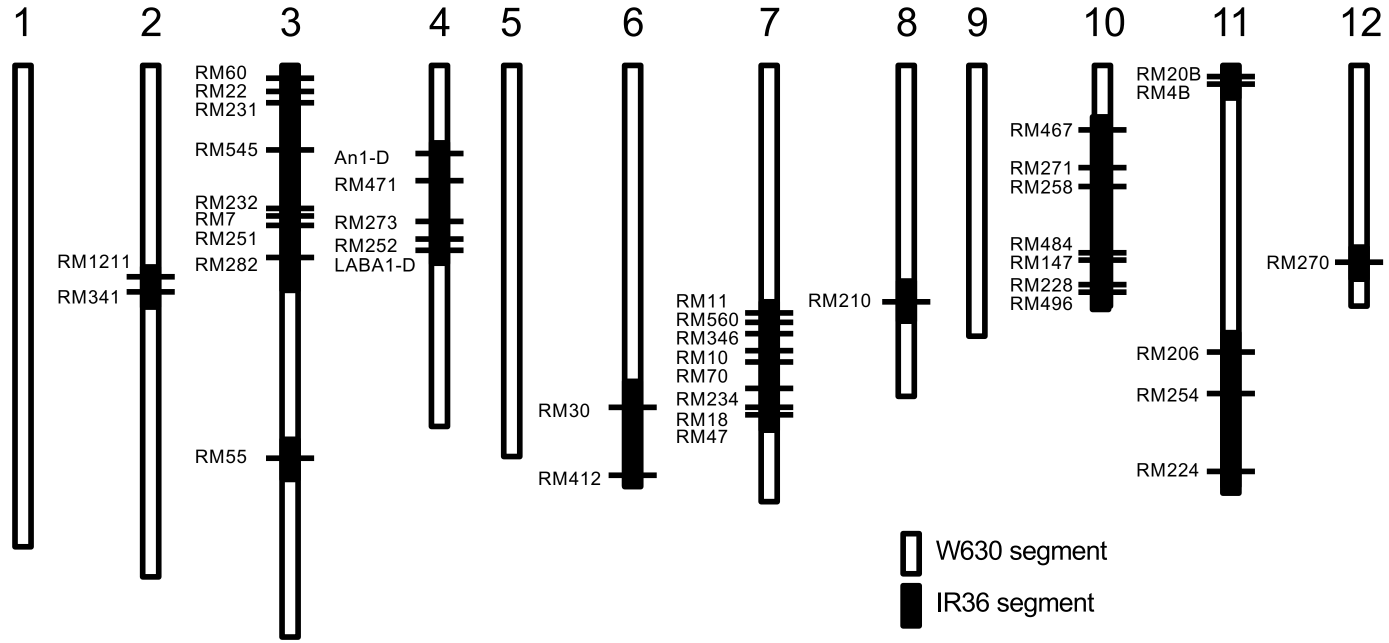

Figure S1 Graphical genotype of the J5 backcross recombinant inbred line.

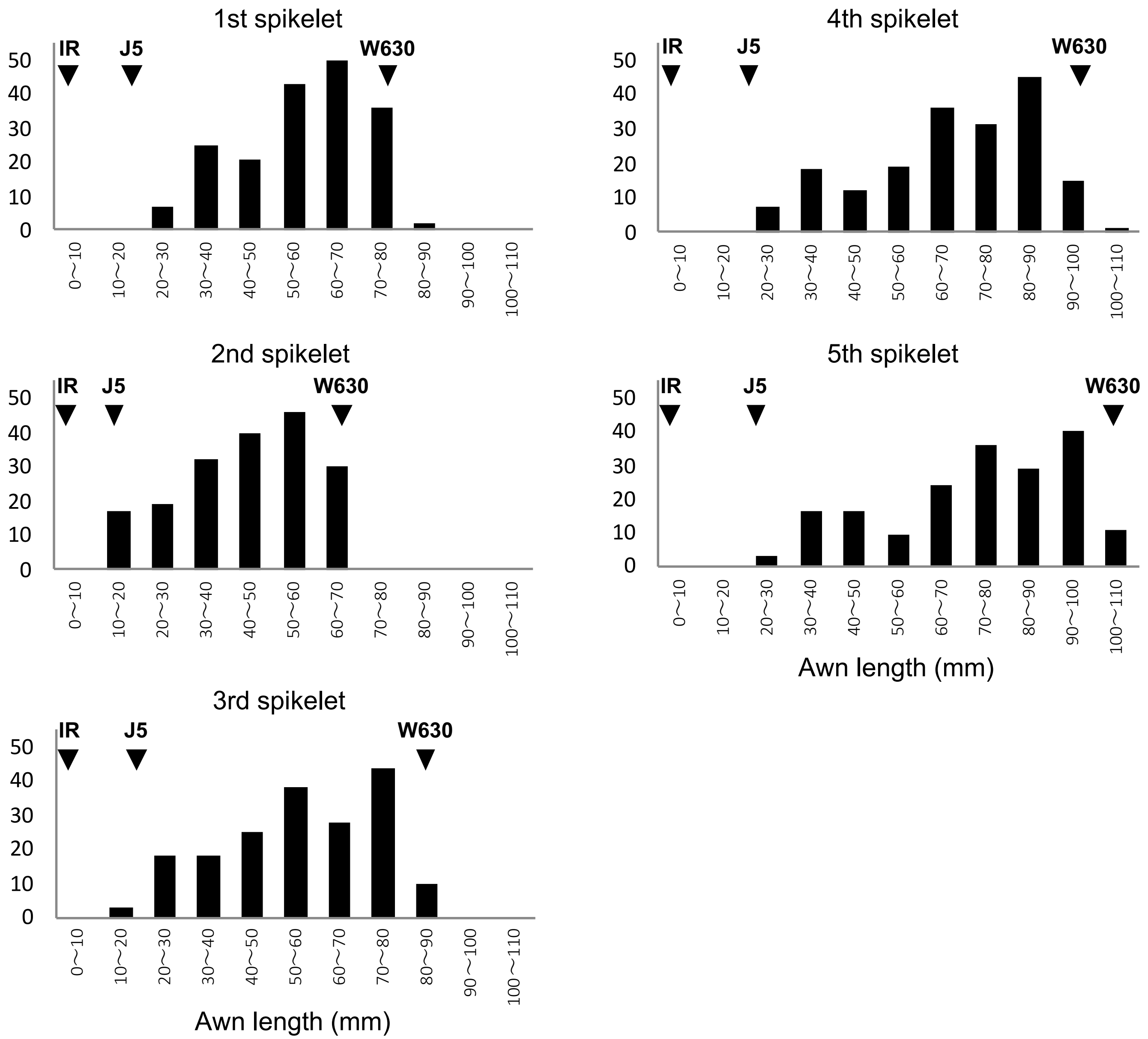

Figure S2 Frequency distributions of awn lengths in the 1st to 5th spikelet positions in BC_3F_2 population between *O. rufipogon* W630 and the J5 line. The triangles show the average values of the parental lines.

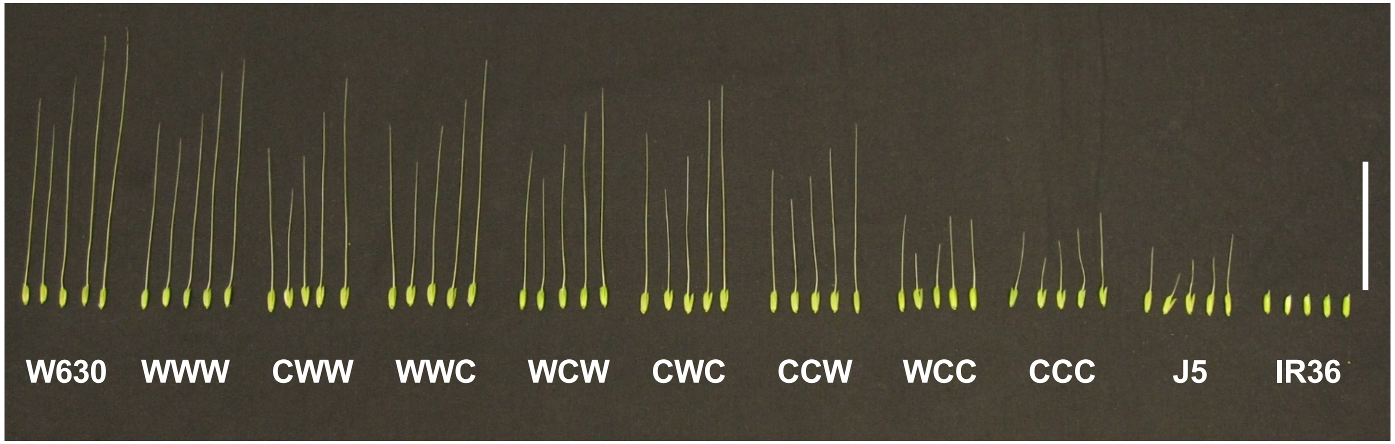

Figure S3 Spikelet morphology of the eight genotypes of BC_3F_3 plants together with *O. rufipogon* W630, J5 line and *O. sativa* IR36. The eight genotypes were designated by three-letter combinations of W or C in genotypic order at *qAWNL2*, *An-1* and *LABA1*, where W and C indicate wild and cultivated homozygous alleles, respectively. For each genotype, the 1st to 5th spikelets are arranged from left to right. Scale bar: 5 cm.

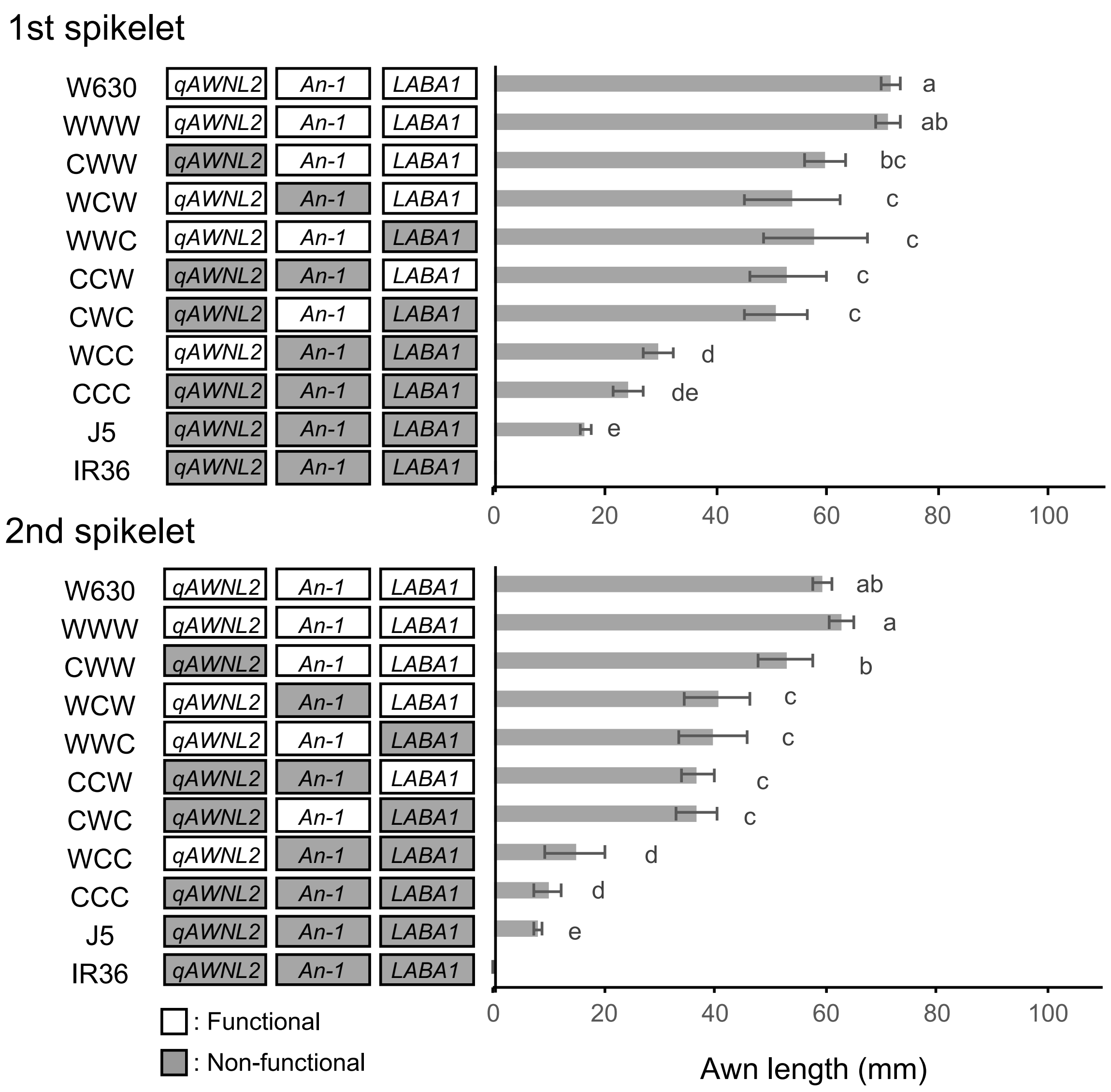

Figure S4 Awn lengths (means ± SD) of the 1st to 4th spikelets of eight homozygous genotypes in the wild genetic background. They are compared together with the parental accessions of *O. rufipogon* W630 and the J5 line. Average awn lengths of *O. sativa* IR36 are shown for reference. Eight genotypes were designated by three-letter combinations of W or C in genotypic order at *qAWNL2*, *An-1* and *LABA1*, where W and C indicate wild and cultivated homozygous alleles, respectively. White and gray boxes with the locus names beside the genotypes indicate functional (wild) and non-functional (cultivated) homozygous alleles, respectively. Means labeled with different letters are significantly different among the eight genotypes and two parental lines (Tukey's test, $P < 0.05$).

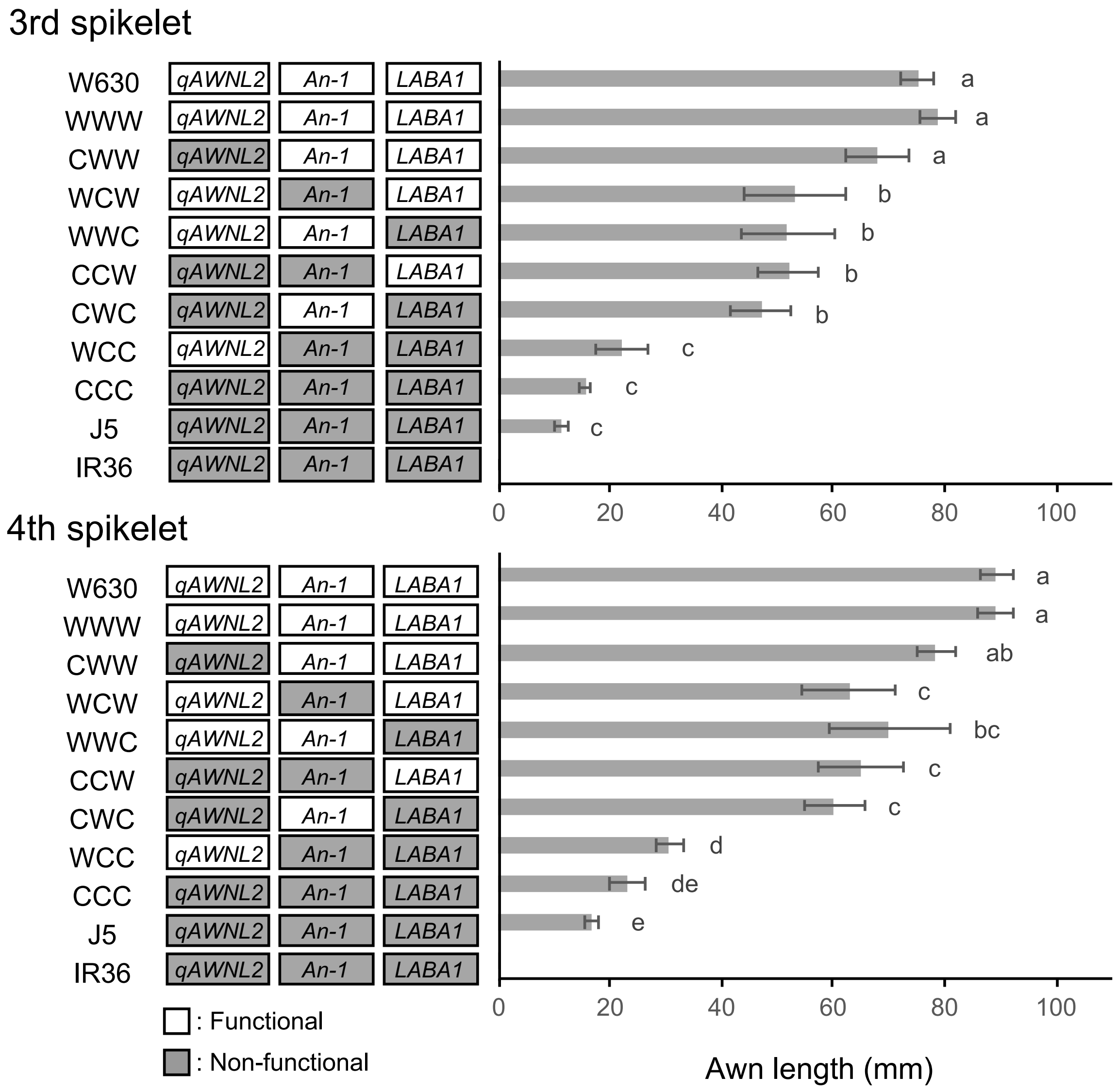

Figure S4 (continued)
